# Anti-Proliferative Effect of Camellianin A in *Adinandra nitida* Leaves and Its Apoptotic Induction in Human Hep G2 and MCF-7 Cells

**DOI:** 10.3390/molecules15063878

**Published:** 2010-05-28

**Authors:** Han Gao, Benguo Liu, Feng Liu, Yongsheng Chen

**Affiliations:** 1 School of Food Science, Henan Institute of Science and Technology, Xinxiang 453003, China; E-Mails: gh@hist.edu.cn (H.G.); chysh11@126.com (Y.C.); 2 The Key Laboratory of Chemical Biology, Graduate School at Shenzhen, Tsinghua University, Shenzhen 518055, China; E-Mail: liu.feng@sz.tsinghua.edu.cn (F.L.)

**Keywords:** *Adinandra nitida*, camellianin A, flavonoid, apoptosis, cell cycle

## Abstract

Leaves of *Adinandra nitida* constitute a kind of flavonoid-rich plant food. In this study, camellianin A, the main flavonoid in the leaves of *Adinandra nitida*, was prepared and identified by high performance liquid chromatography-photodiode array detector-electrospray ionization mass spectrometry (HPLC-PDA-ESI/MS). In the anticancer assay, it was found camellianin A could inhibit the proliferation of the human hepatocellular liver carcinoma Hep G2 and human breast adenocarcinoma MCF-7 cell lines in a dose-dependent manner and induce the significant increase of the G0/G1 cell population. After treated by camellianin A, phosphatidylserine of Hep G2 and MCF-7 cells could translocate significantly to the surface of the membrane. The increase of an early apoptotic population of Hep G2 and MCF-7 cells was observed. It was concluded that camellianin A not only affected the progress of the cell cycle, but also induced cells to enter into apoptosis.

## 1. Introduction

Flavonoids have received great attention and have been studied extensively for their antioxidant, antibacterial, analgesic antitumor properties. It is believed that a higher intake of flavonoid-rich foods is associated with decreased risk of many degenerative diseases, particularly cardiovascular diseases and cancer [1,2].

**Figure 1:**
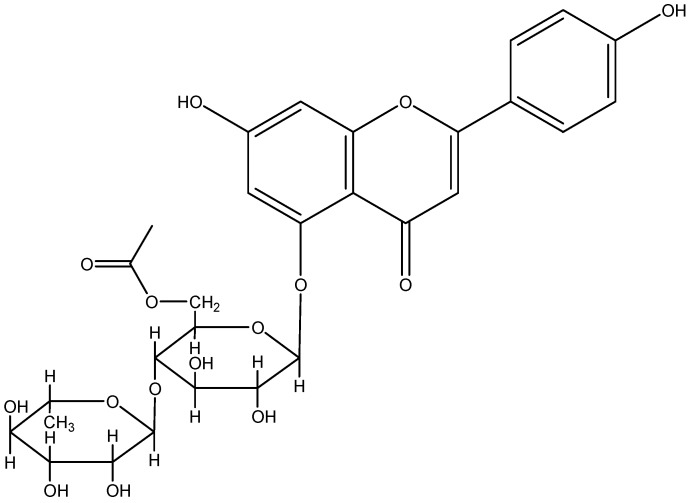
Chemical structure of camellianin A.

*Adinandra nitida* (Theaceae), a wild plant from South China, is a flavonoid-rich plant source. Its leaves have been consumed as a health tea (Shiyacha) and herbal medicine for hundreds of years [3,4]. It is reported to have many curative effects, such as reducing blood pressure, as well as antibacterial, antioxidant, and analgesic properties [5]. According to our previous studies [6,7,8], the total flavonoid content in *A. nitida* leaves can be more than 20%. Nowadays, *A. nitida* can be artificially cultivated in China, so there is the possibility for large-scale use of camellianin A in the health food and phytopharmaceutical industry. However, so far *A. nitida* leaves have not been thoroughly studied. For example, it was always thought that the anticancer activity of *A. nitida* leaves was due to its flavonoids, but this was not supported by any experimental evidence. In this study, camellianin A (Figure 1), the main flavonoid in *A. nitida* leaves, was separated and its apoptotic induction in human Hep G2 and MCF-7 cells were investigated for the first time.

## 2. Results and Discussion

### 2.1. Identification and determination of Camellianin A

**Figure 2:**
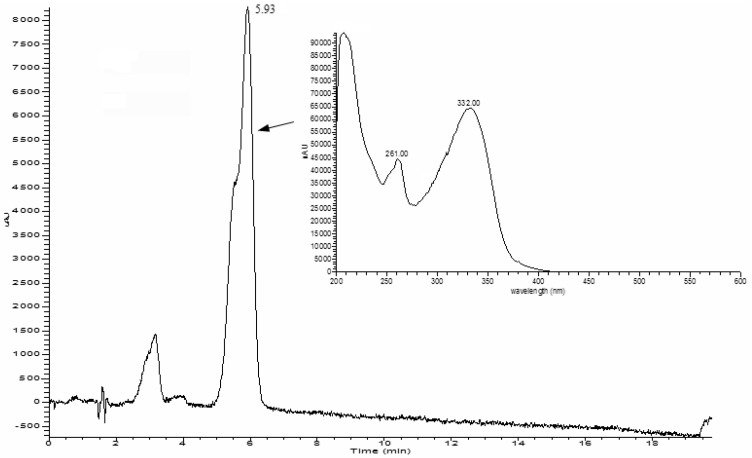
HPLC-PDA profile of camellianin A.

**Figure 3:**
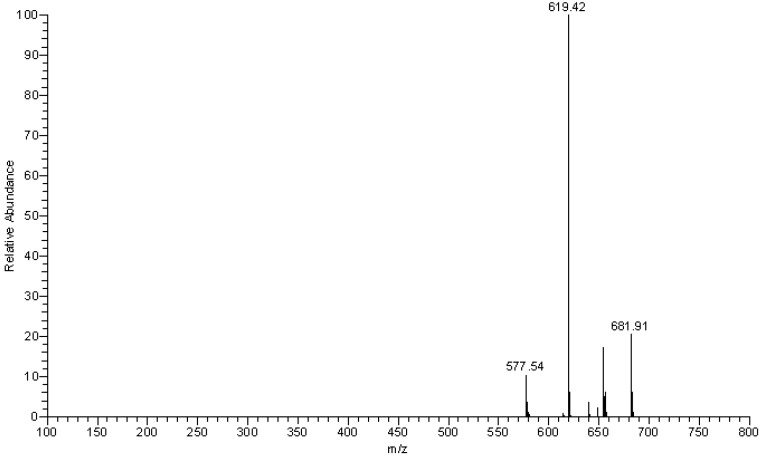
ESI-MS of camellianin A.

**Figure 4:**
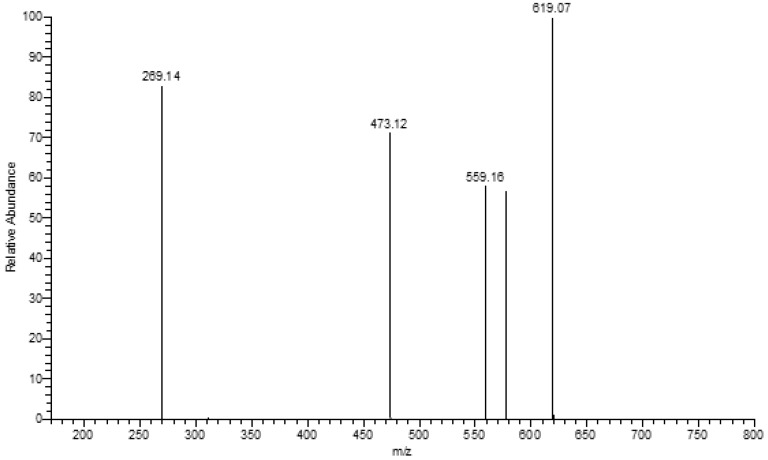
ESI-MS^2^ of camellianin A.

By using HPLC-PDA-ESI/MS, the compound eluted at 5.93 min in the HPLC (Figure 2) was identified as camellianin A. The UV and ESI-MS^n^ data (Figure 3 and Figure 4) were as follows: UV, λ_max_ (nm) 261, 332; ESI-MS^2^ negative ion *m/z*: 681.91 ([M+NO_3_]^-^), 654.87 ([M+Cl]^-^), 619.42 ([M-H]^-^), 577.54 ([M-Acetyl-H]^-^), 473.12 ([M-Rham-H]^-^), 269.14 ([M-Acetyl-Rham-Glu-H]^-^), which coincided with our previous study [6]. The content of camellianin A in *A. nitida* leaves was determined as 19.25 ± 0.24% by HPLC.

### 2.2. Anti-proliferative effect of camellianin A on human Hep G2 and MCF-7 cells

**Figure 5:**
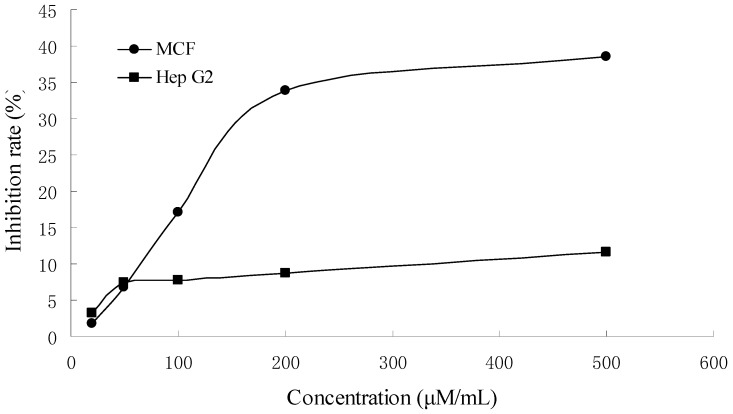
Effect of camellianin A on Hep G2 and MCF-7 cell proliferation.

The inhibitory effects of camellianin A on the proliferation of Hep G2 and MCF-7 cells were tested at different concentrations for 48 h and the inhibition rates were determined (Figure 5).

Camellianin A induced a dose-dependent inhibitory effect. Nearly 33.8%, 8.7% of MCF-7 and Hep G2 cells were inhibited by 200 μM camellianin A. Compared with Hep G2 cells, inhibition of MCF-7 cell proliferation by camellianin A was more effective. The differential activity of camellianin A on both cell lines represented the selectivity of its antitumour action. Though camellianin A could inhibit the proliferation of Hep G2 and MCF-7 cells, there were other compounds present in *A. nitida* leaves which could also contribute to its antiproliferative activity.

### 2.3. Cell cycle analysis

To determine whether the Hep G2 and MCF-7 cells treated with camellianin A undergo the apoptosis pathway, the cell distribution in the cell cycle was examined by PI staining.

**Figure 6:**
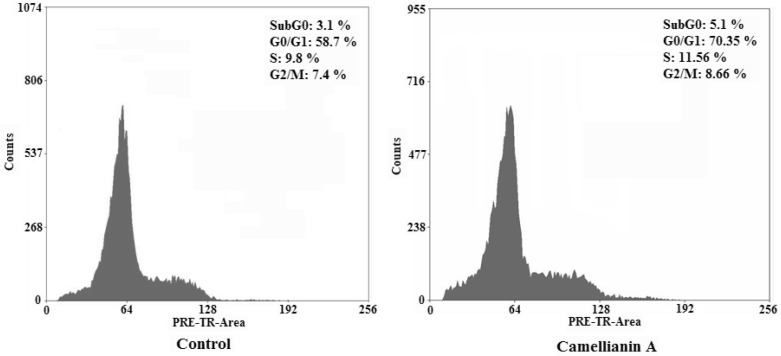
Effect of camellianin A on cell cycle distribution in MCF-7 cells.

**Figure 7:**
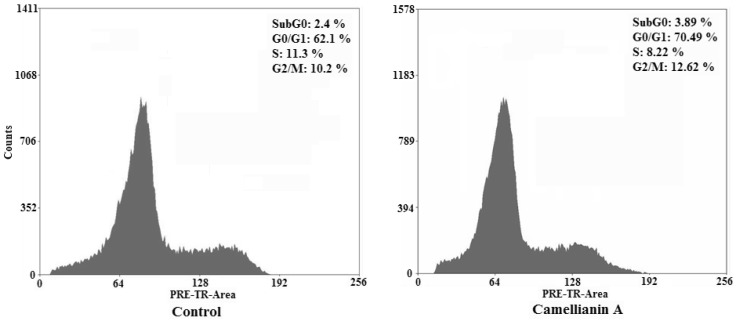
Effect of camellianin A on cell cycle distribution in Hep G2 cells.

Camellianin A induced the increase of the cell population in the cell population in the G0/G1 phase in MCF-7 and Hep G2 cells (Figure 6 and Figure 7). It was found that the G0/G1 population of treated tumor cells increased dramatically which was suggested to be the apoptotic DNA.

### 2.4. Flow cytometry analysis of cell apoptosis

**Figure 8:**
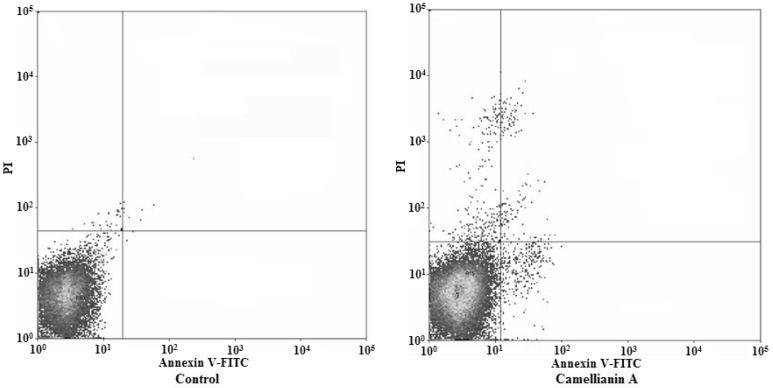
Flow cytometric analysis of phosphatidylserine externalization and cell membrane integrity in MCF-7 cells undergoing apoptosis.

**Figure 9:**
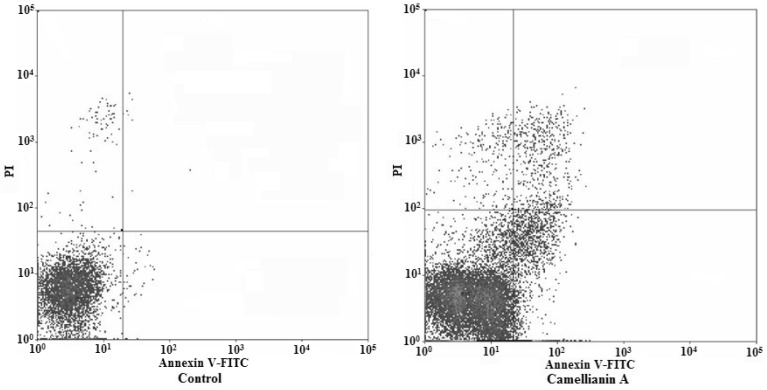
Flow cytometric analysis of phosphatidylserine externalization and cell membrane integrity in Hep G2 cells undergoing apoptosis.

Apoptosis is a highly regulated cell death process with characteristic biochemical features [9,10] and membrane-bond apoptotic bodies [11].The hallmark of early apoptotic cells is the transverse redistribution of plasma membrane phosphatidylserine (PS); thus, the annexin V binding assay was performed to detect the surface exposure of PS. Figure 8 and Figure 9 showed the FACS histograms with dual parameters including annexin V-FITC and PI. 

The dual parametric dot plots combining annexin V-FITC and PI fluorescence showed the viable cell population in the lower left quadrant (annexin V-negative/PI-negative), the early apoptotic cells in the lower right quadrant (annexin V-positive/PI-negative), and the late apoptotic cells in the upper right quadrant (annexin V-positive/ PI-positive). In untreated MCF-7 cells, 0.01% of cells were the early apoptotic cells, 0.1% of cells were the late apoptotic cells. The early and late apoptotic cells increased to 1% and 0.46%, respectively, after being treated with camellianin A at a concentration of 0.5 mM for 48 h. For Hep G2 cells, the control cells, only 0.7% cells were the early apoptotic cells and 2.7% cells were the late apoptotic cells. The early and late apoptotic cells were 15.08% and 2.7%, respectively after the cells were treated with camellianin A at the concentration of 0.8 mM for 48 h. Our work demonstrated that PS could significantly translocate to the surface of the membrane in the Hep G2 and MCF-7 cells. Early apoptotic cells were observed by both annexin V-FITC and PI staining. These result suggested that camellianin A in *A. nitida* leaves could inhibit the proliferation of Hep G2 and MCF-7 cells in a dose-dependent manner and induced the cells to enter into apoptosis. 

## 3. Experimental

### 3.1. Materials and chemicals 

Leaves of *A. nitida* (2009 production, moisture content 9.3%) for this study were purchased in Pingle, China, and identified by Professor Yousheng Zhang in Guangdong Academy of Agricultural Sciences. The voucher specimen was deposited in the Laboratory of Food Chemistry of Henan Institute of Science and Technology. The camellianin A standard was obtained from our previous study. The human hepatocellular liver carcinoma Hep G2 and human breast adenocarcinoma MCF-7 cell lineS were obtained from the Institute of Medicinal Biotechnology, Chinese Academy of Medical Sciences. Powdered Dulbecco modified eagle medium and trypsin solution were purchased from GIBCO (Grand Island, NY, USA). Fetal bovine serum (FBS) and antibiotics (penicillin and streptomycin mixture) were purchased from Hyclone Laboratories, Inc. Propidium iodide (PI) and 3-[(4, 5)-dimethylthiazol-2-yl]-2, 5-diphenyl tetrazolium bromide (MTT) were purchased from Sigma Chemical Co. An annexin V-FITC apoptosis detection kit was purchased from Clontech Laboratories Inc. Other chemicals were of analytical grade.

### 3.2. Preparation of Camellianin A

About 24.38 g of powdered *A. nitida* leaves was extracted with 500 mL of 60% ethanol (v/v) in a water bath at 70 °C for 2 h and then filtered. The filtrate was concentrated under vacuum at 55 °C. The obtained solution was kept for 24 h at room temperature. The filtered precipitate (6.72 g) was collected and purified to obtain the white crystals by recrystallizing three times from 30% ethanol (v/v). After drying, about 3.50 g of the product was gained for identification and anticancer tests.

### 3.3. HPLC-PDA-ESI/MS analysis

A sample of the obtained above powder (10 mg) was dissolved and diluted to 100 mL with methanol. After filtration through 0.45 μm Millipore filter, a 10 μL volume of the solution was injected for HPLC-PDA-ESI/MS analysis, which was performed using an LCQ Deca XP MAX system (Finnigan, USA). Separation was performed on an Eclipse×DB-C18 column (150 × 4.6 mm i.d.; 3.5 μm particle size). The mobile phase consisted of methanol and water (6:4). The flow rate was 0.8 mL/min. The first detection was by Photodiode Array Detector (PDA), and the second detection employed an Electrospray Ionization Mass Spectrometry (ESI-MS). Due to the acidic nature of flavonoids, the mass analyzer was set to record the negative spectra. The scan range was set from 100 to 800 m/z.

### 3.4. Determination of the content of Camellianin A in A. nitida leaves by HPLC 

Dried powdered leaves (1.029 g) were placed in a Soxhlet extractor and refluxed at 80 °C for 10 h with methanol (150 mL) and then the extract was collected and diluted to 500 mL with methanol. After filtered through a 0.45 μm Millipore filter, a 10 μL volume of the solution was injected for HPLC analysis. For the calibration curve, the stock solution of camellianin A standard was diluted with methanol in the concentration range from 28.65 to 573 μg/mL for camellianin A, All the solutions were stored at 4 °C. The sample was separated on a reversed phase column, SunFire^TM^ C18 column (4.6 × 250 mm; 5 μm particle size) made by Waters. The mobile phase consisted of water and methanol (1:1) with a flow rate of 1.0 mL/min. The column temperature was set at 30 °C. The HPLC analysis was performed on a Waters Alliance HPLC system, which consisted of a Waters 2695 separations module and a Waters 2487 dual wavelength detector. The injection volume was 10 μL and the wavelengths for detection were set at 265 nm and 330 nm. The quantitative analysis of camellianin A in the sample was based on an external standard. The chromatographic data were recorded and processed by Empower 2 software.

### 3.5. Cell culture and drug treatment 

The human MCF-7 and Hep G2 cells were cultured in DMEM medium with 10% FBS, 100 UI/mL penicillin and 100 μg/mL streptomycin in humidified air at 37 °C with 5% CO_2,_ respectively. The exponentially growing cells were collected and re-suspended in fresh medium for 4 h and then exposed to various concentrations of camellianin A.

### 3.6. MTT assay 

Survival of cells was evaluated by using a system based on MTT, which was reduced by living cells to yield a soluble formazan product that could be detected colorimetrically. Cells were suspended in 96-well plates of 90 μL medium at a density of 2 × 10^4^ cells/well and 10 μL camellianin A at different concentrations. These were then incubated in humidified air at 37 °C with 5% CO_2_ for 48 h, exposed to 10 μL MTT (5 mg/mL) and incubated for another 4 h under the conditions mentioned above. The formazan precipitate was dissolved in 100 μL DMSO. IC_50_ values were tested through the MTT method [12].The inhibition rate was calculated as follows: Inhibition Rate % = (mean control absorbance-mean experimental absorbance)/mean control absorbance × 100%.

### 3.7. Flow cytometry analysis

The flow cyctometry analysis was performed on a FACS Calibur Flow cytometer (BeckmanCoulter, USA). Cell pellets were fixed in 70% ethanol at –20 °C for at least 12 h or overnight. After being washed twice with ice-cold PBS, they were incubated in RNase A/PBS (1 mg/mL) at 37 °C for 30 min, and stained with PI (0.5 mg/mL) at room temperature for 15 min. The intracellular DNA was then labeled with PI and the PI fluorescence of individual nuclei determined by a FACSCalibur fluorescence-activated cell sorter at 488 nm excitation. Surface exposure of phosphatidylserine in apoptotic cells was measured by the annexin V-FITC apoptosis detection kit according to the manufacturer’s instructions. Additional exposure to PI made it possible to differentiate the early apoptotic cells (annexin V-positive/PI-negative) from the late apoptotic cells (annexin V-positive and PI-positive).

### 3.8. Statistical analysis

The data obtained in this study were expressed as the mean of three replicate determinations and standard deviation (SD). Statistical comparisons were carried out using student t test. P values of <0.05 were considered to be significant.

## 4. Conclusions

Though flavonoids exist ubiquitously in plants, few plant species contain the levels needed to achieve large-scale production. *Adinandra nitida* leaves are rich in camellianin A. In this study, it was found that that camellianin A could inhibit the proliferation of the human hepatocellular liver carcinoma Hep G2 and human breast adenocarcinoma MCF-7 cell lines in a dose-dependent manner by inducing cells to enter into apoptosis. The obtained result could be the basis for its alleged health promoting potential of *Adinandra nitida* leaves and *A. nitida* leaves could serve as a new source of natural nutraceuticals with potential applications in the food and medicine industries.

## References

[1] Burda S., Oleszek W. (2001). Antioxidant and antiradical activities of flavonoids. J. Agric. Food Chem..

[2] Zou Y., Lu Y., Wei D. (2004). Antioxidant activity of a flavonoid-rich extract of *Hypericum perforatum* L. *in vitro*. J. Agric. Food Chem..

[3] Wang Y., Chen S.B., Ni J., Yao X., Ye W.C., Zhao S.X. (2003). Chemical studies on the Adinandra nitida (In Chinese). J. China Pharm. Univ..

[4] Zhang J., Yang J., Duan J., Liang Z., Zhang L., Huo Y., Zhang Y. (2005). Quantitative and qualitative analysis of flavonoids in leaves of *Adinandra nitida* by high performance liquid chromatography with UV and electrospry ionization tandem mass spectrometry detection. Anal. Chim. Acta.

[5] Yu J., Chen M. (1997). Studies on flavonoids extraction from Adinandra nitida Merr. ex H. L. Li. and on their antioxidative and bacteriostatic bioactivities (in Chinese). J. Shantou Univ..

[6] Liu B., Ning Z., Zhan Y., Xu K., Gao J. (2008). Characterization and 1, 1-diphenyl-2-picrylhydrazyl radical scavenging activity of methanol and supercritical carbon dioxide extracts from leaves of *Adinandra nitida*. J. Food Biochem..

[7] Yuan E., Liu B., Ning Z. (2008). Preparation and antioxidant activity of camellianin A from *Adinandra nitida* leaves. J. Food Process Pres..

[8] Yuan E., Liu B., Ning Z., Chen C. (2009). Preparative separation of flavonoids in *Adinandra nitida* leaves by high-speed counter-current chromatography and their effects on human epidermal carcinoma cancer cells. Food Chem..

[9] Jacobson M.D., Weil M., Raff M.C. (1997). Programmed cell death in animal development. Cell.

[10] Nagata S. (1997). Apoptosis by death factor. Cell.

[11] Kidd V.J. (1998). Proteolytic activities that mediate apoptosis. Annul. Rev. Physiol..

[12] Mosmann T. (1983). Rapid colorimetric assay for cellular growth and survival: Application to proliferation and cytoxicity assays. J. Immunol. Method..

